# Microbial Dynamics: Assessing Skincare Regimens’ Impact on the Facial Skin Microbiome and Skin Health Parameters

**DOI:** 10.3390/microorganisms12122655

**Published:** 2024-12-21

**Authors:** Nicole Wagner, Valerie Diane Valeriano, Samuel Diou-Hirtz, Evelina Björninen, Ulf Åkerström, Lars Engstrand, Ina Schuppe-Koistinen, Johanna Maria Gillbro

**Affiliations:** 1 Center for Translational Microbiome Research, CTMR, Department of Microbiology, Tumor and Cell Biology (MTC), Science for Life Laboratory, Karolinska Institute, 171 65 Solna, Sweden; valerie.valeriano@ki.se (V.D.V.); samuel.diouhirtz@ki.se (S.D.-H.); evelina.bjorninen@ki.se (E.B.); lars.engstrand@ki.se (L.E.); ina.schuppe.koistinen@ki.se (I.S.-K.); 2 Skinome Research AB, Hornsgatan 172, 117 28 Stockholm, Sweden; ulf@skinome.se

**Keywords:** microbiology, skin microbiome, dermatology, 16S rRNA, microbiome analysis, effects of skincare

## Abstract

The human skin microbiome, a complex ecosystem of microbes, plays a pivotal role in skin health. This study aimed to investigate the impact of two skincare regimens, with preservatives (CSPs) and preservative-free (PFPs), on the skin microbiome in correlation to skin quality. double-blind randomized cosmetic studywith a split-face design was conducted on 26 female participants. Microbial diversity and abundance were analyzed using 16S rRNA amplicon sequence data and skin quality utilizing the Antera 3D skin camera. We confirmed earlier studies on the identification of major skin microbial taxa at the genus level, including *Cutibacterium acnes*, *Corynebacterium*, and *Neisseriaceae* as a predominant part of the facial skin microbiome. Furthermore, microbiome profile-based subgrouping was employed, which revealed that the cluster, characterized by the Neisseriaceae family as its predominant organism, exhibited significant reduction in folds count, fine lines, and redness after application of PFP compared to CSP. A Spearman correlation analysis highlighted the correlation between changes in specific bacteria and skin quality parameters such as redness, pores, and texture in the context of comparing PFP and CSP. Overall, the PFP treatment demonstrated a greater number of significant correlations between bacterial changes and skin quality compared to the CSP treatment, suggesting a distinct impact of the preservative-free skincare regimen on the skin microbiome and skin quality. Our study provides insights into different microbiome-centered approaches to improve our understanding of the skin microbiome’s interplay with skin quality but also highlights the need for larger, comprehensive research to further understand the microbiome’s role in dermatology.

## 1. Introduction

The skin microbiome has recently gained significant attention in scientific research. Focusing on the human skin microbiome, particularly for dermatological and cosmetic purposes, is emerging as a critical field to investigate for promoting optimum skin health [1,2,3,4].

The skin microbiome is an intricate ecosystem, housing trillions of microbes, including around a thousand species, predominantly bacteria [5]. A diverse and balanced microbiome is closely linked with healthy skin [1,6,7]. This balance is maintained by host factors and the interactions among various microorganisms, helping to prevent colonization by pathogenic bacteria [8,9]. Most microbes on the human skin are commensals, living symbiotically by utilizing by-products from host cells and, in return, aiding in maintaining the skin’s low pH to prevent pathogen colonization as well as improving skin hydration [10,11,12].

Research indicates a connection between decreased microbial diversity and skin disorders such as acne vulgaris, psoriasis, rosacea, and atopic dermatitis [3,13,14,15]. For instance, atopic dermatitis is characterized by a higher presence of *Staphylococcus aureus,* which contributes to the aggravation of symptoms [16].

Sebaceous glands are essential for maintaining skin homeostasis, as they release oils that protect the epidermis [17]. These secretions create a lipid-rich microenvironment that supports the growth of commensal bacteria, including *Cutibacterium acnes*. The complexity of this microenvironment is further underscored by the interaction between sebaceous gland regeneration and hair follicle stem cells. This regenerative process, mediated by β-catenin signaling, replenishes the sebaceous gland, highlighting the role of stem cells in sustaining the microenvironment where *C. acnes* thrive [18].

Although *C. acnes* has been implicated in conditions such as acne vulgaris, research suggests that acne is more closely associated with a loss of *C. acnes* phylotype diversity rather than its overgrowth [19]. While these processes provide insights into the ecological niche of *C. acnes*, our study focuses on healthy skin, where this bacterium is abundant and contributes to the dynamic balance of the human skin microbiota.

The appearance of the skin is closely tied to overall well-being, as dermatological conditions can profoundly impact patients’ daily lives, self-esteem, mental health, and social interactions due to their visibility [20]. Studies have reported a high prevalence of psychiatric and psychosocial comorbidities, such as anxiety and depression, among individuals with skin disorders. While the psychological effects of skin disorders on appearance are well documented, even non-pathological changes in skin appearance can have a significant impact. This underscores the importance of studying healthy skin to better understand its dynamics and the factors that influence its appearance.

Despite the increasing number of studies exploring the microbiome’s role in disease states, comparatively less effort has been directed toward understanding its impact on normal skin and factors like skin aging. However, recently, Myers et al. investigated the role of the skin microbiome on skin aging, and the authors showed that a decline in microbial diversity was linked with reduced skin barrier function, as indicated by increased trans-epidermal water loss (TEWL). Also, *Actinomycetes* were linked to high facial wrinkling in contrast to key commensal Gram-positive bacteria associated with lower facial wrinkling and higher hydration levels [21].

However, fewer studies have been conducted that explore the effects of external factors on the skin microbiome. For example, people growing up in urban environments are exposed to more sterile conditions compared to people growing up closer to nature, being more exposed to environmental microorganisms [22]. Skincare products also seem to affect the skin microbiome and skin chemistry, and compounds from beauty products can build up in the skin even after frequent washing, affecting the skin’s microbiome in the long term [23]. Preservatives are antimicrobial ingredients used in cosmetics to prevent microbial growth, provide product stability, and ensure a long shelf life, with a standard of more than 30 months. Broad-spectrum preservative efficacy is required to meet the cosmetic regulations, and, therefore, several different preservatives are added to cosmetic products to prevent microbial growth. However, the preservatives are not only active in the product but are also active on the skin long after they have been applied and can, therefore, influence the skin commensal microbes. It is still relatively unknown how chemicals, especially the preservatives, used in personal care products influence the skin microbiome. It has been shown that the commonly used preservatives inhibit and kill residential *C. acnes* and *Staphylococcus epidermidis* of the skin [24]. In another in vitro study by Wang et al., the maximum allowed amounts of the most used preservatives in cosmetics did inhibit the survival of all nine skin-residential flora isolated from healthy adults [25]. Several studies also indicate that beneficial skin bacteria seem to be more sensitive to preservatives than the pathogens [26]. Additionally, in vivo studies have shown that traditional cosmetic products, all containing conventional preservatives, affect the skin microbiome [27,28]. This was confirmed by Santa-Maria et al., where it was shown that freshly made skincare without preservatives significantly increased the diversity of the skin microbiome [1].

The aim of the current study was to identify microbial taxa of interest associated with skin quality signs using taxonomic profiling, initially assigning 1449 species consisting of 16S rRNA amplicon sequence data.

The study was conducted by exploring the impact of two distinct skincare regimens on the skin microbiome, one incorporating preservatives (CSPs) and the other devoid of such additives (PFPs), via a “split face” clinical design that included 26 female participants to compare the effects before and after the application of the two different routines for three weeks.

Here, we present methods to investigate microbial taxa and the strength of response to different treatments based on clusters of individuals constructed based on diversity and the dominant organisms present in the skin microbiome profile in relation to skincare treatment and skin quality using the S Illumina MiSeq platform with V3 chemistry as well as taxonomic profiling.

## 2. Methods

### 2.1. Subject Population

Twenty-six healthy Caucasian volunteers (27–44 years old) with normal skin and no history of skin disease were enrolled. The majority were regular skincare users. Exclusion criteria included diagnosed skin disease, pregnancy, and nursing. The cosmetic study adhered to the Declaration of Helsinki and good clinical practice, approved by the Swedish Ethical Review Authority (Dnr 2022-07196-01).

### 2.2. Study Design

This double-blind randomized cosmetic studyused a split-face design. Participants applied different skincare products to each side of their face: conventional skincare products (CSPs) on one side and products without traditional preservatives (PFPs) on the other. Treatments were randomized and blinded. Participants applied moisturizers in the morning and evening, cleansing before evening application, for three weeks in March–April 2023. Skin microbiome and biophysical parameters were assessed at the start and after three weeks.

### 2.3. Composition of Test Products According to INCI

- PFP Cleanser: Aqua, Cetearyl Alcohol, Stearic Acid, Glycerin, Kaolin, Coco-Betaine, Polyglyceryl-3 Dicitrate/Stearate, C10-18 Triglycerides, Lactobacillus Ferment, Avena Sativa Kernel Oil, Caprylic/Capric Triglyceride, Sodium Methyl Cocoyl Taurate, Squalane, Niacinamide, Dextrin, Polydextrose, Amylopectin, Sodium Cocoyl Isethionate, Salicylic Acid.
- PFP Facial Cream: Aqua, Jojoba Esters, Squalane, Vegetable Oil, Glycerin, Hydrogenated Vegetable Oil, Stearyl Alcohol, Glyceryl Stearate, Lactobionic Acid, Urea, Phytosterol, Ceramide NP, Zinc PCA, Calcium PCA, Sodium Stearoyl Glutamate, Tocopherol, Sodium Polyacryloyldimethyl Taurate, Sodium Hydroxide.
- CSP Cleanser: Aqua, Sodium Laureth Sulfate, Decyl Glucoside, Glycerine, Sodium Chloride, Coco-Betaine, Salicylic Acid, PEG-150 Pentaerythrityl Tetrastearate, PEG-6 Caprylic Glycerides, Zinc Gluconate, Sodium Hydroxide, Capryloyl Salicylic Acid, Tetrasodium EDTA, Citric Acid, Menthol, Polyquaterium-47, Hexylene Glycol, Sodium Benzoate.
- CSP Facial Cream: Aqua, Caprylic/Capric Triglyceride, Propanediol, Glycerin, Silica, Cetearyl Alcohol, Dipentaerythrityl Tetrahydroxystearate/Tetraisostearate, Glyceryl Stearate, PEG-100 Stearate, Phenoxyethanol, Cetearyl Glucoside, Cetyl Alcohol, Perilla Ocymoides Seed Oil, Stearyl Alcohol, Dimethicone, Xylitylglucoside, Fragrance, Polyacrylamide, Anhydroxylitol, C13-14 Isoparaffin, Ethylhexylglycerin, Xylitol, Butylene Glycol, Dimethiconol, Tocopherol, Glyceryl Acrylate/Acrylic Acid Copolymer, Decylene Glycol, Disodium EDTA, Laureth-7, Diospyros Mespiliformis Leaf Extract, Glucose, Maltodextrin, Kalanchoe Pinnata Leaf Extract, Sanicula Europaea Extract, Tromethamine, Citric Acid, Lapsana Communis Flower/Leaf/Stem Extract, Furcellaria Lumbricalis Extract, Sodium Benzoate, Potassium Sorbate, Maris Sal/Sea Salt/Sel Marin, Callicarpa Japonica Fruit Extract.

### 2.4. Sample Collection

Skin swabs were taken from both cheeks of 26 participants, collected in DNA/RNA shield (Zymo Research, Orange, CA, USA). Participants applied CSP to one side and PFP to the other. After three weeks, swabs were collected again from each cheek.

### 2.5. Biophysical Measurement of Skin Quality

Skin biophysical parameters were assessed using the Antera 3D camera (Miravex Limited, Ireland), which captured comprehensive images of skin color, redness, pigmentation, texture, volume, fine lines, and wrinkles. The measurement area was 56 × 56 mm, with data processed using the Antera Pro software, version 3.1.10 2023 [28,29,30,31,32,33].

### 2.6. Extraction

Samples were thawed, transferred to ZR BashingBead lysis tubes (Zymo Research, Irvine, CA, USA), and subjected to bead beating using a FastPrep24 5G (MP Biomedicals, Santa Ana, CA, USA). After centrifugation, the samples were purified using the ZymoBIOMICS 96 MagBead DNA protocol and stored at −20 °C.

### 2.7. Sequencing

DNA quantity was assessed using the Quant-iT 1X dsDNA Assay kit (Thermo Fisher Scientific, Waltham, MA, USA). The samples were standardized to 170 ng and amplified using 341F/805R primers. The PCR products were purified, combined, normalized, and sequenced on the MiSeq platform with V3 chemistry (Illumina, Inc., San Diego, CA, USA).

### 2.8. Bioinformatic Analysis

All bioinformatic analyses were conducted utilizing QIIME2-2023.2 [29] and R 4.2.2 [30].

*Quality control*

The demultiplexed paired-end Fastq reads underwent initial quality control and trimming using the DADA2 plugin in Qiime2. Forward sequences were truncated at 260 bp, while reverse sequences were truncated at 220 bp, in order to retain only the highest confidence base assignments. Following this, the forward and reverse reads were merged using the DADA2 plugin. Reads below Q20 (1 error in 100) were removed. A total of 16 individuals’ sequences successfully passed the DADA2 denoise-paired quality check, and these samples were subsequently utilized for the following analysis. Two individuals were later removed when it seemed that, based on their profiles, the second time point of the CSP treatment was switched between the two. A total of 14 samples remained for further analysis.

*Taxonomic profile*

The taxonomic profiling was conducted using the QIIME feature-classifier and classify-sklearn plugins with the Silva 138 database [31]. Initially, 1449 species were assigned. The QIIME output underwent cleaning by excluding any organism with fewer than 100 reads in total across all samples, resulting in 382 retained organisms. Subsequently, the values were normalized for each sample to yield a relative abundance for each organism within the respective samples.

### 2.9. Statistical Analysis

*Change in Microbiome*

The central log-ratio (CLR) of the normalized samples was computed using the CLR function from the R Composition package version 2.0-8 [32]. The alteration in the microbiome between the two time points (TP1 and TP2) was assessed by computing the change in the CLR value between the two time points. This transformation facilitates the exploration of relative changes in abundance while mitigating the impact of compositional constraints.

*Microbiome Profile-Based Subgrouping*

Considering the variation in taxonomic profiles among the samples across different subject groups, the subjects were grouped into five clusters using Ward’s method based on their taxonomic composition at the species level. One cluster consisted of only a single individual (subject 23) and was consequently excluded from the cluster analysis. The analysis focused on the remaining four clusters to explore correlations between subject subdivisions and the Antera variables.

*Alpha Diversity Comparison Between Treatments*

The R Vegan package version 2.6-4 [33] was employed for the analyses, encompassing exploration of alpha diversity metrics within each treatment group and highlighting differences between treatments. The measured alpha diversity metrics included Shannon, inverse Simpson, and richness values.

Alpha diversity was calculated to assess changes between treatments within each individual. Additionally, alpha diversity was computed for individuals at each time point and treatment within different clusters.

For beta diversity, specifically dissimilarity between groups, the Jensen–Shannon Divergence (JSD) method from the Vegan package [29,33] in R was utilized to examine differences between samples. The Jensen–Shannon Divergence serves to compare the distribution of microbial taxa across different samples.

In calculating the Jensen–Shannon divergence, we initially computed the Kullback–Leibler divergences between each distribution and the average distribution. These individual divergences were then averaged, and the square root of the result was obtained. The resulting JSD values provided a quantitative measure of dissimilarity, with higher values indicating greater divergence.

*Antera Data Changes for Combined Samples and Clusters*

The alteration in Antera variables was evaluated by measuring the difference between the two samples, with a negative value indicating a decrease in the category between the two time points and a positive value indicating an increase in the measurement at the second time point.

To determine the statistical significance of the changes in the Antera variables between the two time points, the Wilcoxon test was employed. This analysis was conducted for both the entire set of individuals combined and individuals within each cluster, aiming to investigate whether the taxonomic composition and diversity of the group influences their response to the treatments.

*Significant Organismal Changes in Antera Variables with Treatment, Incorporating Bias Correction*

The “ancombc2” function from the ANCOMBC2 package [34] in R was utilized to identify any significantly differentially abundant organisms between the samples. The Benjamini–Hochberg (BH) method was used for *p*-value bias correction. The analysis was conducted to assess significant abundance changes of organisms between the two treatments, incorporating time points and subjects for bias correction.

*Spearman Analysis for Correlation Assessment*

Spearman values were computed for the change in Antera variables and change in microbiome using the “cor.test” function in the R 4.2.2 base package [30]. Subsequently, the BH (Benjamini–Hochberg) method was used to correct for FDR (False Discovery Rates). The organisms with a significant correlation for each Antera variable were then extracted, and the “rho value” indicating correlation between the taxa and the variables was analyzed. The Spearman analysis aimed to identify changes in the metadata correlation with the change in microbial abundance.

## 3. Results

### 3.1. Taxonomic Profile

**Figure 1:**
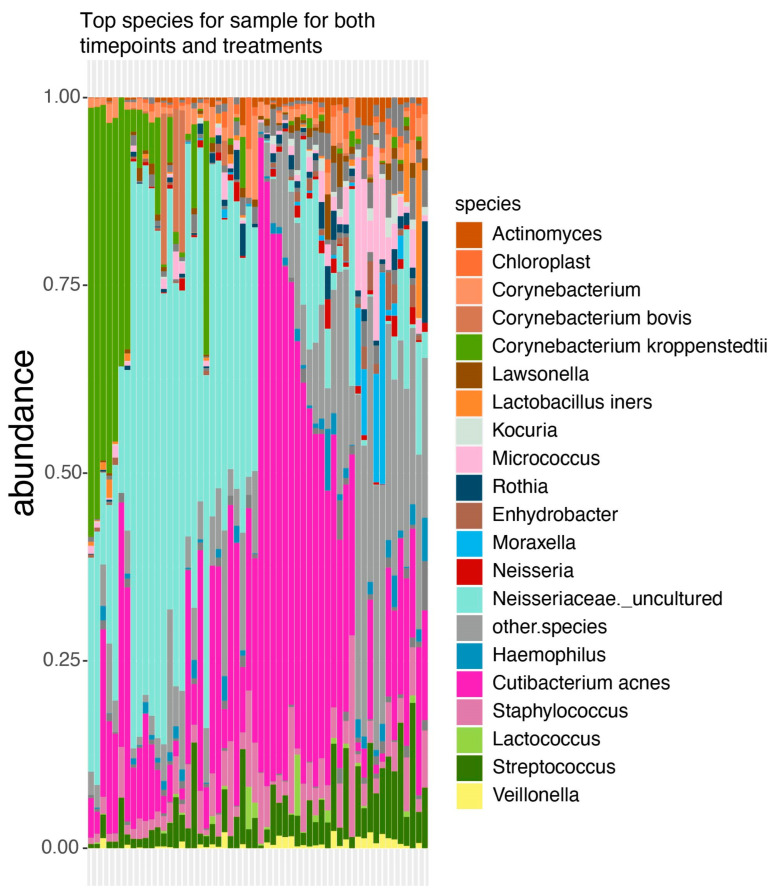
Taxonomic profile of all samples, including the top 20 samples, with the remaining being denoted as “other species”.

The analyzed samples exhibited distinct microbial abundance profiles, although some displayed notable similarities. Mainly *Corynebacterium kroppenstedii*, *C. acnes*, and an uncultured bacterium within the family Neisseriaceae emerged as the predominant species. While most samples featured one of these key organisms as the dominant species, sample 23 had a markedly distinct profile, characterized by the prevalence of *Moraxella* and *Micrococcus*. Additionally, sample 23 demonstrated higher diversity, with nearly half of its microbial profile comprising the top 20 species, whereas the remaining samples primarily consisted of these top organisms (Figure 1).

### 3.2. Clustering of Samples Based on Ward’s Method

**Figure 2:**
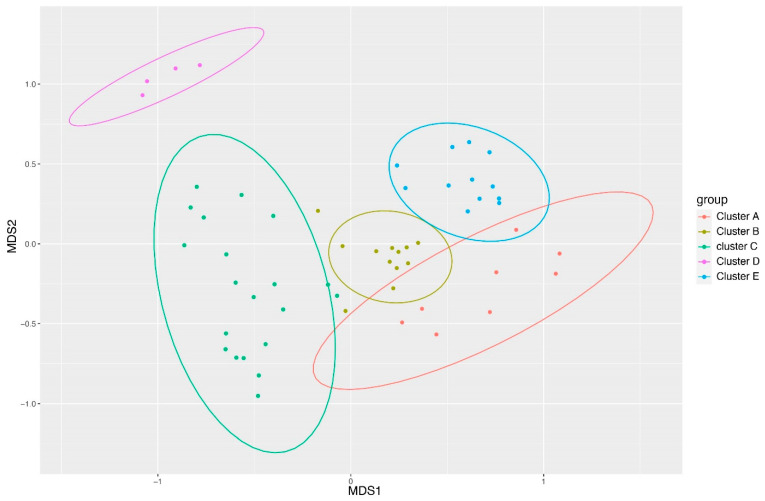
NMDS profile of the different clusters of samples showing that the separation can be seen clearly with cluster D, representing sample 23 as being farthest from the other samples.

Given discernible differences in taxonomic profiles, the Ward’s method was employed for sample clustering using similarity or dissimilarity metrics. Common metrics include Jaccard similarity and Bray–Curtis dissimilarity. Ward’s method hierarchically agglomerates samples, aiming to minimize the total within-cluster sum of squares. Both pre- and post-treatment samples for CSP and PFP clustered together for each sample, suggesting that while the microbiome undergoes changes, these alterations do not fundamentally transform the skin microbiome. A Non-Metric Multidimensional Scaling (NMDS) analysis corroborated Ward’s clustering output, revealing distinct clusters, with subject 23 grouping separately from all other groups (Figure 2).

### 3.3. Taxonomic Profile per Cluster

**Figure 3:**
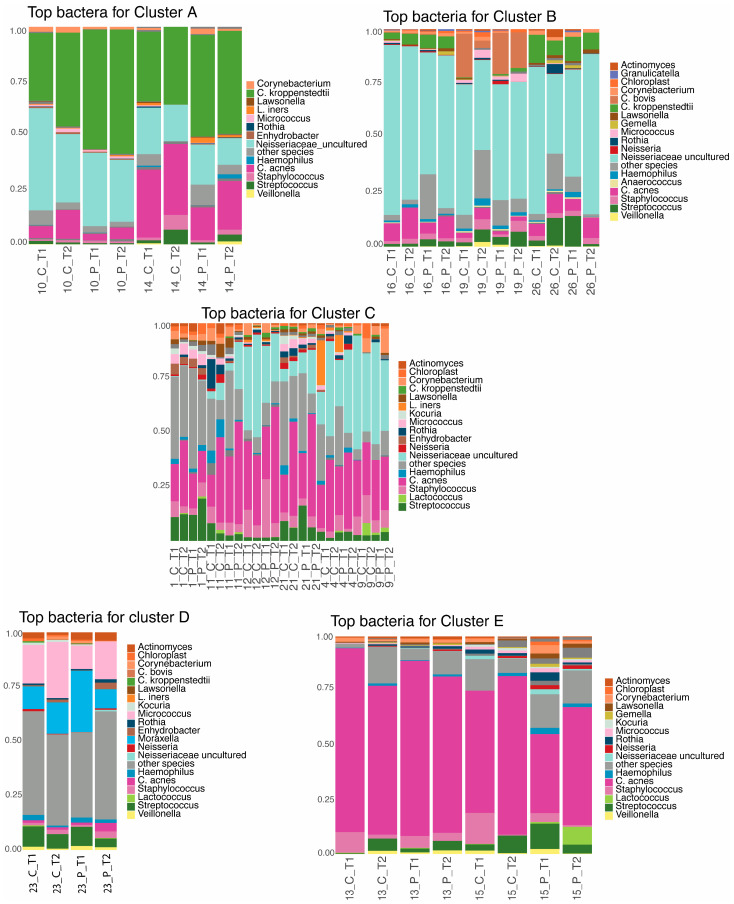
Taxonomic profile of the top 20 species, along with “other species” in each cluster.

For each cluster, taxonomic profiles focusing on the top 20 species were generated. Clusters D and, to a lesser extent, C showed higher proportions of “other species”, suggesting potentially higher alpha diversity within these clusters (Figure 3).

### 3.4. Alpha Diversity Before and After Treatment

**Figure 4:**
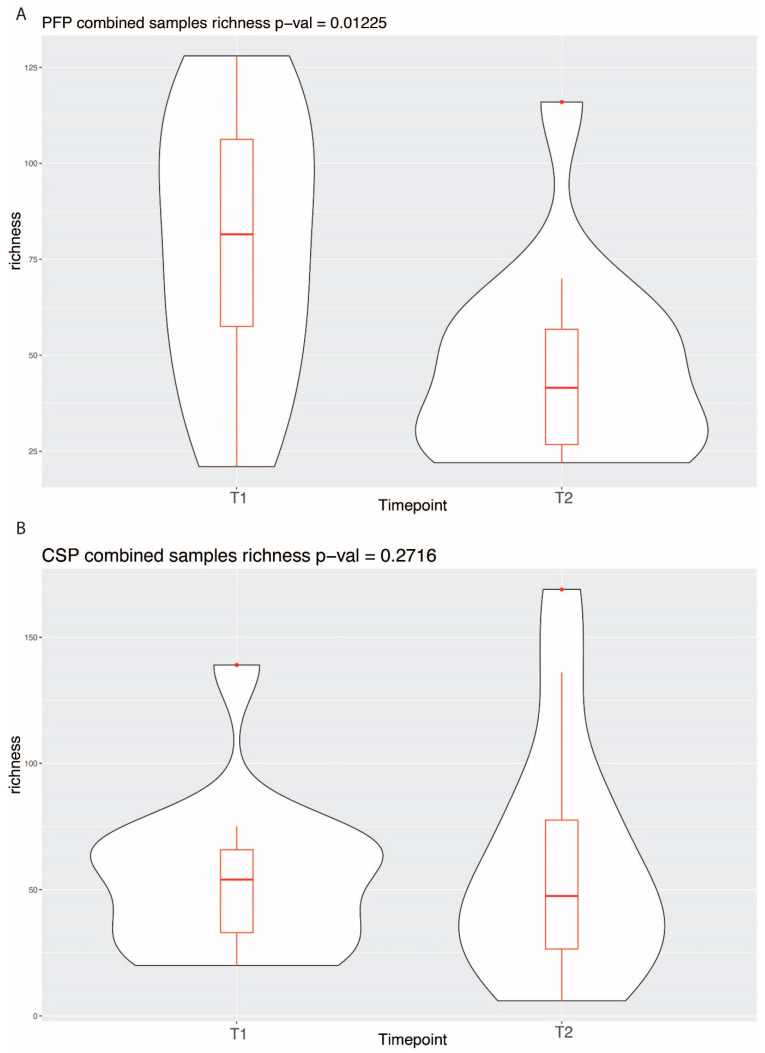
Alpha diversity between time point 1 and time point 2 for each treatment.

Alpha diversity was computed using richness, Shannon, and inverse Simpson metrics. While no significant differences were observed in the Shannon and inverse Simpson indices, the richness index showed distinctive patterns. The richness index quantifies the number of distinct species within a sample. As can be seen in Figure 4, while no significant change in richness was found between pre- and post-treatment communities in the CSP treatment, the PFP treatment demonstrated a statistically significant decrease in richness post-treatment (*p*-value: 0.01).

### 3.5. Alpha Diversity Between Clusters

**Figure 5:**
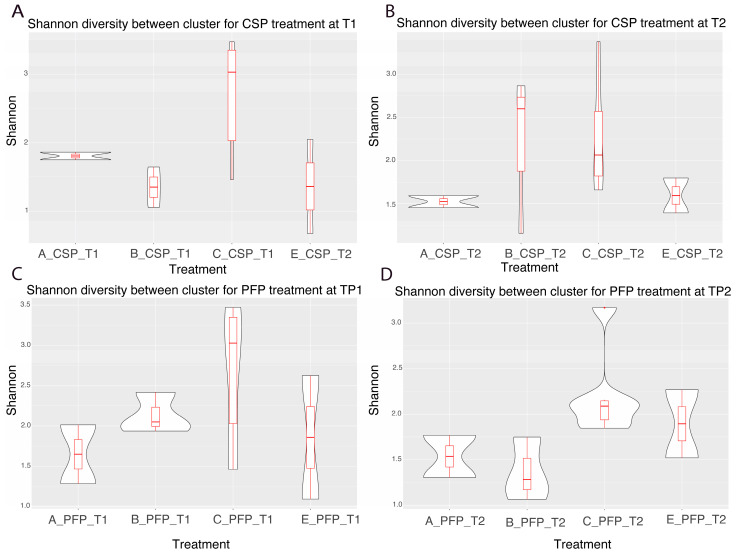
Shannon diversity between clusters at different points in the treatment.

**Figure 6:**
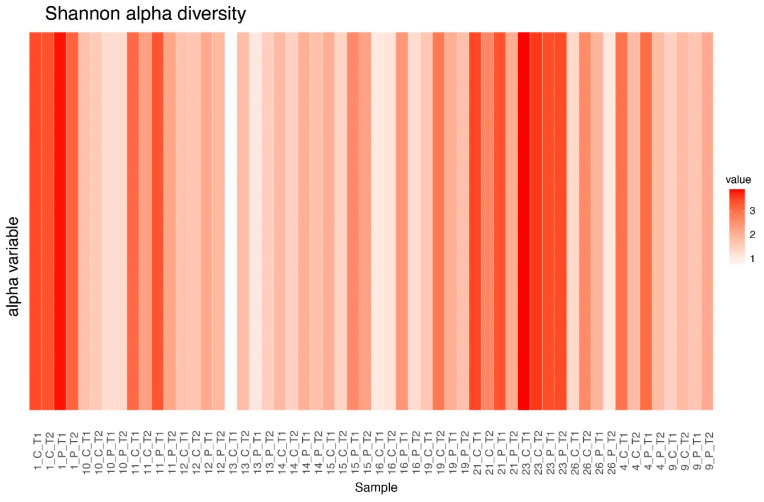
Shannon diversity difference values for each of the samples.

Shannon alpha diversity, providing information on both richness and evenness of communities, was examined across clusters at different treatment stages. A significant difference in alpha diversity was observed between cluster C and other clusters before treatment, but this significance diminished post-treatment for both CSP and PFP conditions. No significant difference between clusters was observed in the post-PFP treatment. Cluster D was excluded from this analysis as it only contained sample 23 (Table 1, Table 2, Table 3 and Table 4; Figure 5 and Figure 6).

### 3.6. DeltaCLR of Species (For Top 10 Decreased and Top 10 Increased Species)

**Figure 7:**
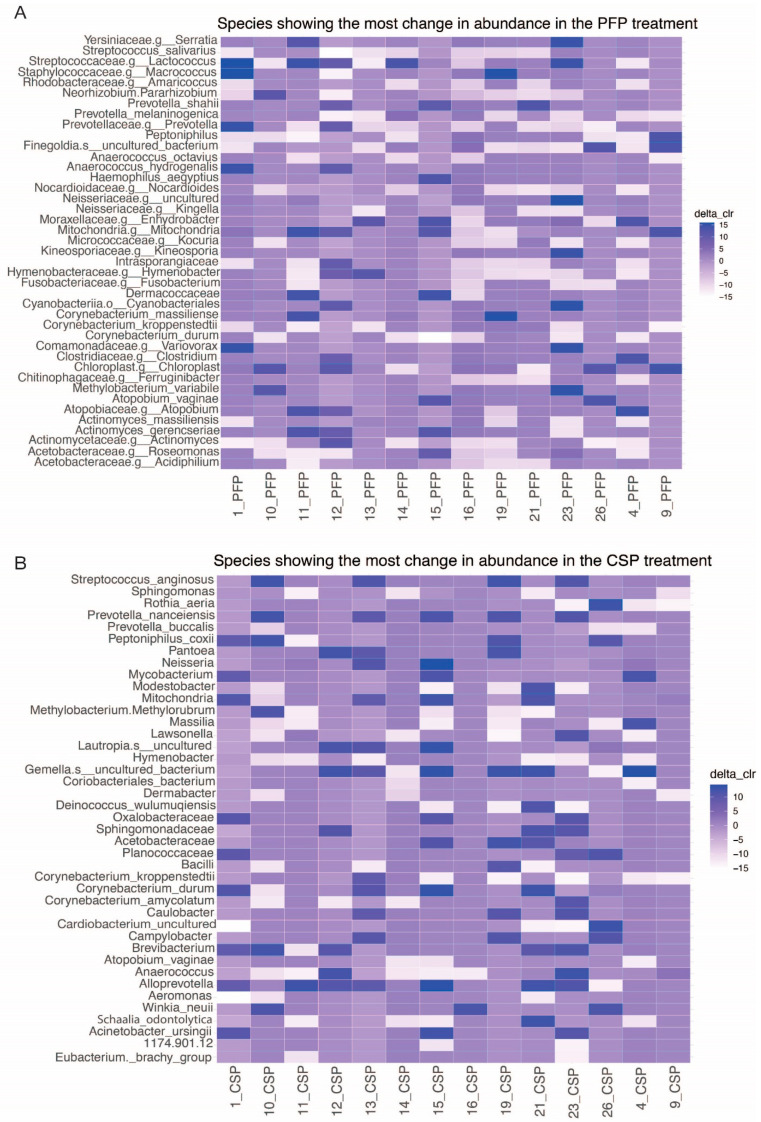
Heatmaps depicting the delta CLR (the lighter color indicates a decrease after treatment, and the darker color indicates an increase after treatment).

The Delta CLR method evaluated changes in microbial abundance between time points, identifying log-ratio differences in microbial composition. Positive values indicate an increase, while negative values signify a decrease in the abundance of a species between the two time points. *Corynobacterium kroppenstedii* increased under both treatments. In the PFP treatment, changes were noted in *Neisseriaceae* and *Actinomyces*, whereas the CSP treatment showed changes in *Prevotella*. The observed changes did not show a consistent trend across treatments and subjects (Figure 7).

### 3.7. Jensen–Shannon Beta Diversity

**Figure 8:**
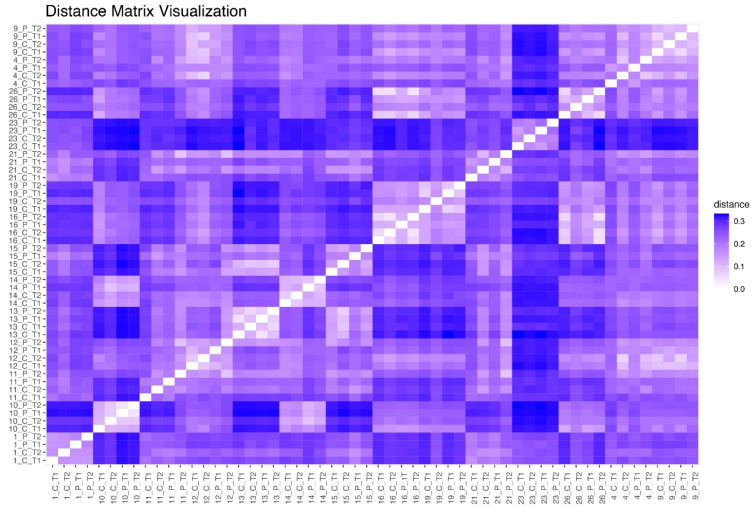
Jensen–Shannon Distance between the samples.

Jensen–Shannon Divergence (JSD) was used to quantify dissimilarity between taxonomic profiles. Smaller differences were observed between samples within clusters A, B, and E, with the highest dissimilarity reaching 0.3. The largest dissimilarity (0.35) was observed between sample 23 (cluster D) and samples in other clusters (Figure 8).

### 3.8. Differential Abundance Analysis Using ANCOMBC2

**Figure 9:**
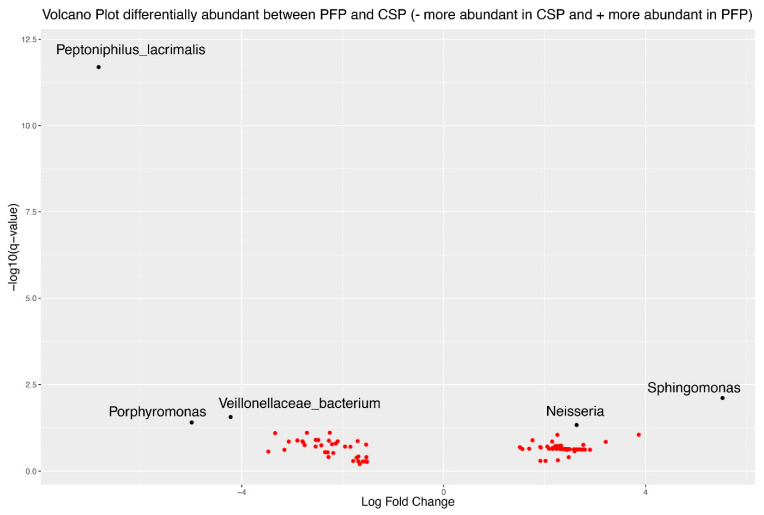
ANCOMBC2 volcano plot indicating the log-fold change on the *x*-axis and log10 of FDR-corrected *p*-value (q-value) on the *y*-axis. Black dots represent organisms with a significantly different log fold change of value greater than absolute value of 2.

ANCOMBC2 (Analysis of Compositions of Microbiomes with Bias Correction 2) was used to investigate differential abundance between the PFP and CSP treatments. Among the 95 organisms with a log-fold change greater than 1.5, 5 exhibited an FDR-corrected statistically significant difference. *Sphingomonas* and *Neisseria* increased in the PFP treatment, while Porphyromonas, *Peptoniphilus lacrimalis*, and *Veillonellaceae* were more abundant in the CSP treatment (Figure 9).

### 3.9. Comparison of Change Between Treatments

**Figure 10:**
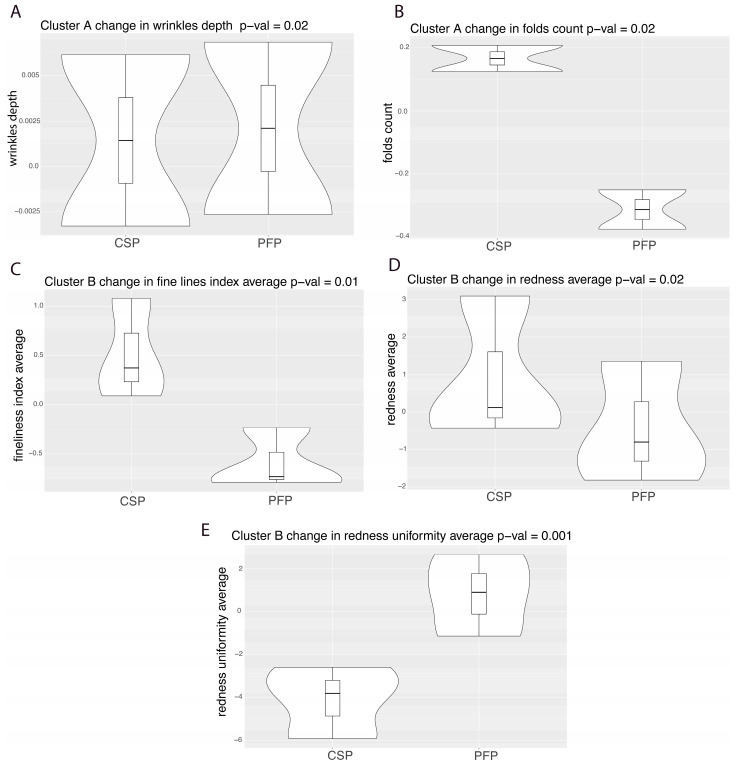
Significant changes observed in cluster 2 between the amounts of change in the two treatments for the variables.

Significant changes were observed between time points, with notable changes in clusters A and B. Cluster A showed significant changes in wrinkle depth and fold count, while cluster B exhibited significant alterations in fine lines, redness, and redness uniformity (Figure 10).

### 3.10. Spearman Correlation Analysis

**Figure 11:**
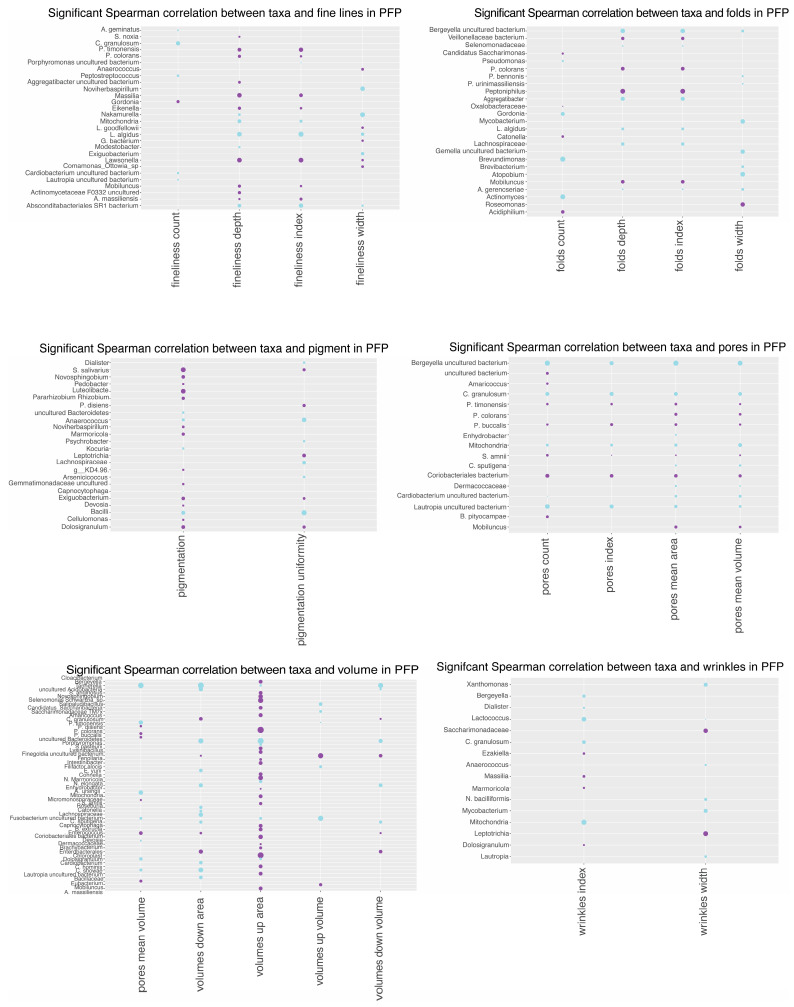
Significant Spearman correlation between taxa and Antera variables for the PFP treatment. Purple dots signify a positive correlation, while blue ones signify a negative correlation.

**Figure 12:**
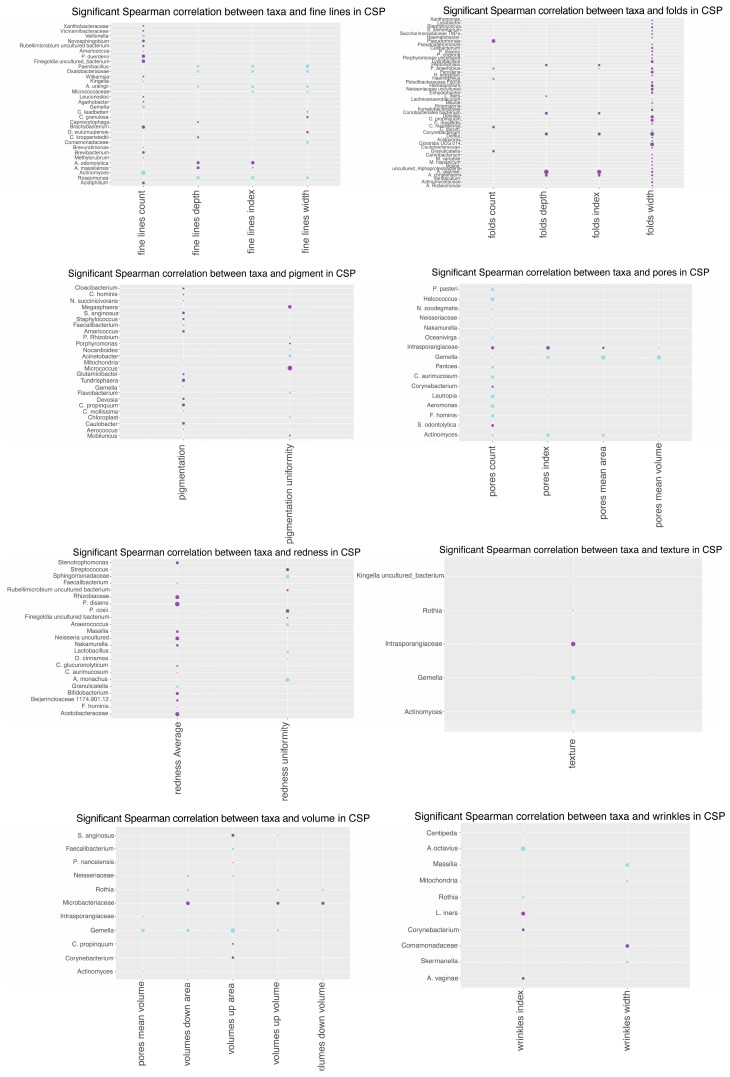
Significant Spearman correlation between taxa and Antera variables for the CSP treatment. Purple dots signify a positive correlation, while blue ones signify a negative correlation.

Spearman correlation coefficients were computed to quantify relationships between skin microbiome changes and Antera variables. The PFP treatment exhibited more significant correlations with bacteria compared to CSP. Positive and negative correlations were observed, indicating relationships between microbial abundance changes and skin parameters, such as folds, pigmentation, pores, redness, texture, and volume. The analysis suggests different bacterial responses to the PFP and CSP treatments, with PFP showing more significant correlations in multiple skin parameters (Figure 11 and Figure 12).

The study provides an in-depth analysis of the relationship between specific bacteria and various skin parameters in the context of comparing PFP and CSP skincare treatments.

## 4. Discussion

This study embarked on an exploratory journey to investigate the interactions between skincare regimens and the skin microbiome, leveraging a side-by-side comparison of conventional skincare products (CSPs) and preservative-free products (PFPs). Our analysis revealed a complex microbial landscape, underlining the significant role of skincare products in shaping the skin’s microbial milieu.

Consistent with prior investigations [1,35,36,37], *C. acnes*, *Corynebacterium*, and *Neisseriaceae* were identified as the principal taxa within our samples. Notably, while *Corynebacterium* is acknowledged as part of the commensal skin microbiome, especially in moisture-rich areas, like the armpit, our findings uniquely identify *C. kroppenstedtii* as a dominant species in the healthy facial skin microbiome, diverging from earlier research. This is particularly interesting given Li et al.‘s [38] discovery of ethnicity’s role in shaping the skin microbiome composition, where the presence of *Corynebacterium* sp. varied among Hispanic, Caucasian, and East Asian populations. In their study, *C. kroppenstedtii* was specifically identified in East Asians. Our study, which exclusively involved Caucasian participants, highlights the complexity of the skin microbiome and suggests that both genetic and environmental factors influence its composition. However, it is important to note that the small sample size limits the strength of our conclusions.

Conducting an ANCOMBC2 analysis, we identified 95 organisms with significant log-fold changes, among which five showed statistically significant differences. *Sphingomonas* and *Neisseria* increased after 3 weeks of use of PFP. Interestingly, both *Sphingomonas* and *Neisseria* have lately gained attention due to their beneficial role in skin health.

*Sphingomonas* has shown potential protective roles against UV radiation. A study highlighted that *Sphingomonas* can significantly reduce reactive oxygen species levels in human keratinocytes, suggesting it may help protect the skin from solar damage [39]. In addition, *Sphingomonas* extract has been shown to have significant anti-aging effects on the skin. It notably reduces cellular senescence by inhibiting β-galactosidase activation and the expression of cell cycle inhibitors p21 and p16, concomitantly enhancing skin structure by increasing versican and fibrillin-1 expression [40]. Regarding research related to *Neisseria*, it was recently shown that thermal water’s therapeutic effects on the skin were associated with significant increases of mostly non-pathogenic *Neisseria*, suggesting a positive microbiome shift concomitant with an increase in beneficial bacteria, like *Deinococcus*, which is known to inhibit *S. aureus* and is associated with decreased inflammation. *Rothia mucilaginosa*, known for its anti-inflammatory properties, also increased. Conversely, *Paracoccus* species and *Pseudomonas* decreased [41].

Another interesting study reveals a decrease in *Neisseria* in late bed-timers, which was associated with a decrease in skin hydration content, skin firmness, and elasticity, while TEWL, sebum, and wrinkles significantly increased [42], further suggesting the positive aspect of *Neisseria* as part of the resident skin flora.

With regards to the effect of the two different skincare regimens on the skin microbiome in correlation with skin quality parameters such as pore size, redness, pigmentation, texture, and signs of aging, it was difficult to obtain a clear view on this due to the low number of panelists.

The utilization of microbiome profile-based subgrouping in this study facilitated a more structured analysis of complex microbiome data, providing valuable insights into how clusters, characterized by the predominance of certain bacteria, could influence skin quality parameters. Notably, cluster A, characterized by co-predominance of the *Neisseriaceae* family, and cluster B, characterized by the predominance of the *Neisseriaceae* family, exhibited significant reduction in folds count, fine lines, and redness and improved the uniformity of redness under the PFP treatment compared to the CSP treatment. This suggests that the PFP regimen may influence the composition of the skin microbiome, thereby enhancing skin aesthetics and health.

Notably, the PFP treatment showed a broader array of bacteria with significant positive and negative correlations across various skin quality parameters, suggesting a more pronounced impact on the skin microbiome’s composition and its interaction with the skin.

Further, a Spearman analysis of the specific bacterial correlations revealed that *Actinomyces* and *Gamella* exhibited negative correlations with pore parameters in the CSP treatment, implying that a decrease in the abundance of these bacteria might be associated with an increase in pore size and pore visibility.

*Intrasporangiaceae* showed a positive correlation with pore parameters, indicating that an increase in the abundance of this bacteria can be associated with an increase in the pore parameters being investigated.

*Prevotella* species demonstrated significant correlations with various skin parameters in the PFP treatment, including pores and volume, suggesting their involvement in skin hydration and structural integrity.

With regards to skin texture, the *Gamella* genus correlated positively in the CSP treatment, while several *Prevotella* species correlated with texture improvements in the PFP treatment, highlighting the complex role of the microbiome in skin surface characteristics.

A significant number of organisms correlated with redness in the PFP treatment, suggesting that this regimen may help manage skin inflammation or sensitivity. For wrinkles, more bacteria correlated with changes in the PFP treatment, indicating potential anti-aging effects through microbiome modulation.

This study focused on skin characteristics, but the gut–skin axis also plays a significant role in skin health. Evidence suggests that intestinal dysbiosis may trigger systemic inflammation, disrupting skin homeostasis [43]. Exploring this connection further would provide valuable insights into microbiome dynamics and skin health.

## 5. Conclusions

This study underscores the intricate relationship between the skin microbiome and specific skin health parameters, highlighting how skincare treatments can influence the skin microbiome, potentially offering a microbiome-centered approach to skincare and dermatological health. Despite the lack of significant change in microbiome diversity with the treatments, the utilization of microbiome profile-based subgrouping characterized by the predominance of certain bacteria revealed significant improvement in skin quality parameters for the PFP treatment. This study also confirmed earlier findings on the predominant bacteria as part of the commensal facial microbiome.

However, the study’s small sample size (14 panelists with high-quality data) and short duration (three weeks) limit the generalizability and long-term applicability of the findings. The focus on healthy Caucasian women restricts applicability to other demographics, and potential confounding factors, like diet and lifestyle, were not controlled.

These limitations underscore the need for larger, more comprehensive studies involving diverse participant groups and longer follow-up periods to draw robust conclusions regarding the impact of skincare regimens on the skin microbiome. Such studies are crucial to validate our findings, further elucidate the microbiome’s role in skin health, and guide the development of microbiome-centered skincare treatments.

## Figures and Tables

**Table 1:** Shannon index significance between clusters for CSP pre-treatment. * indicates significant difference. Comparisons with *p*-value are considered significant.

CSP T1	A	B	C	E
A	***-***	***No***	Yes *	***No***
B	***No***	***-***	Yes *	***No***
C	Yes *	Yes *	***-***	Yes *
E	***No***	***No***	Yes *	***-***

**Table 2:** Shannon index significance between clusters for CSP post-treatment. * indicates significant difference. Comparisons with *p*-value are considered significant.

CSP T2	A	B	C	E
A	-	No	No	Yes *
B	No	-	No	No
C	No	No	-	Yes *
E	Yes *	No	Yes *	-

**Table 3:** Shannon index significance between clusters for PFP pre-treatment. * indicates significant difference. Comparisons with *p*-value are considered significant.

PFP T1	A	B	C	E
A	-	No	Yes *	No
B	No	-	Yes *	No
C	Yes *	Yes *	-	No
E	No	No	No	-

**Table 4:** Shannon index significance between clusters for PFP post-treatment.

**PFP T2**	**A**	**B**	**C**	**E**
A	-	No	No	No
B	No	-	No	No
C	No	No	-	No
E	No	No	No	-

## Data Availability

The original contributions presented in the study are included in the article, further inquiries can be directed to the corresponding authors.
